# FOXQ1 promotes endometriosis development by enhancing mitochondrial function

**DOI:** 10.1186/s40659-026-00698-7

**Published:** 2026-07-07

**Authors:** Yu Liu, Mei Chi, Yan Lei, Jing Jin, Xiaojing Lei, Shu Zhang, Qingmei Guan, Ling Zhang, Xin Du

**Affiliations:** 1 Department of Gynecology, Maternal and Child Health Hospital of Hubei Province, Tongji Medical College, Huazhong University of Science and Technology, Wuhan, 430070 P. R. China; 2 Institute of Reproductive Health, Tongji Medical College, Huazhong University of Science and Technology, Hubei Province Wuhan, China; 3 Department of Gynecology, The Third Hospital of Wuhan, Hubei Province Wuhan City, China

**Keywords:** FOXQ1, Endometriosis, Mitochondrial function, Invasion, Migration

## Abstract

**Background:**

Endometriosis (EMs) is a pathological condition characterized by the ectopic growth of endometrial tissue outside the uterine cavity. This study aimed to investigate the role of FOXQ1 in the pathogenesis of EMs, particularly through its regulation of mitochondrial function.

**Methods:**

Eutopic endometrial, ovarian EMs, pelvic sidewall EMs, and bowel EMs samples were obtained from 76 patients undergoing laparoscopic or hysteroscopic surgery. The expression levels of FOXQ1 in EMs tissues were determined using reverse transcription-quantitative PCR (RT-qPCR) and western blot analysis. Furthermore, the mRNA expression levels of pro-inflammatory cytokines, including interleukin (IL)-6, interleukin (IL)-1β, tumor necrosis factor (TNF)-α, and interleukin (IL)-8, were quantified in FOXQ1 overexpression (OE) and FOXQ1 knockdown (siFOXQ1) groups using RT-qPCR. Mitochondrial function in these groups, using primary human endometrial epithelial cells (hEECs), was evaluated by assessing mitochondrial DNA (mtDNA) content, ATP production, reactive oxygen species (ROS) levels, oxygen consumption rate (OCR), and MitoTracker staining. The in vitro effects of FOXQ1 on EMs were further examined in hEECs by analyzing cellular apoptosis, proliferation, invasion, and migration.

**Results:**

The expression of FOXQ1 was markedly elevated in endometriotic lesions, such as those on the pelvic sidewall and bowel, compared to control samples, with localization specifically observed in endometrial epithelial cells. Functional assays conducted on hEECs demonstrated that overexpression of FOXQ1 inhibited apoptosis, upregulated critical inflammatory cytokines (IL-6, IL-1β, TNF-α, IL-8), and promoted cellular proliferation, invasion, and migration. These effects appear to be mediated by enhanced mitochondrial function, as indicated by increased mtDNA content, ATP production, ROS levels, OCR, and MitoTracker signal. Conversely, the group treated with siFOXQ1 exhibited the opposite effects. The receiver operating characteristic curve analysis revealed that the area under the curve for FOXQ1 was 0.8784.

**Conclusions:**

FOXQ1 facilitates the progression of endometriosis by enhancing mitochondrial function, which subsequently stimulates inflammatory responses and promotes critical cellular processes such as proliferation, invasion, and migration.

**Supplementary Information:**

The online version contains supplementary material available at https://doi.org/10.1186/s40659-026-00698-7.

## Introduction

Endometriosis (EMs) is a chronic inflammatory condition marked by the ectopic implantation and proliferation of functional endometrial tissue outside the uterine cavity. This process is driven by intricate pathophysiological mechanisms, including oxidative stress, immune dysregulation, and hormonal imbalances [1, 2]. The etiology of EMs is multifaceted, and the molecular mechanisms responsible for its initiation and progression are not yet fully understood. Among its various subtypes, ovarian endometriosis (OEM) is notably prevalent, primarily due to its significant tendency for ovarian invasion.

FOXQ1, a member of the FOX transcription factor family, encodes a protein that is broadly expressed in various cell types and plays a pivotal role in the regulation of gene expression, the cell cycle, cell proliferation, and differentiation. Numerous cancer studies [3], have validated that FOXQ1 functions as a critical oncogene, participating in tumor proliferation, inflammation, migration, and invasion [4] by activating multiple signaling pathways such as LAMB3/WNT/β-catenin [5], EGFR [6], and PI3K/Akt [7]. Wang et al. found that high FOXQ1 expression is markedly associated with increased the size of epithelial ovarian cancer tumors size, advanced stage, and poor overall survival [8]. Given that endometrial cells in EMs share hallmark features with cancer cells—including proliferative, invasive, and metastatic potential—we hypothesized that FOXQ1 might also play a critical role in EMs pathogenesis.

A critical requirement for sustained cellular proliferation and invasion is an adequate energy supply, a function predominantly fulfilled by mitochondria. Beyond their role as cellular “energy factories” responsible for ATP production, mitochondria are pivotal regulators of metabolism, redox balance, and apoptosis [9]. In EMs, mitochondrial dysfunction has been documented, paradoxically accompanied by characteristics of heightened energy metabolism, excessive reactive oxygen species (ROS) generation, and an increased oxygen consumption rate (OCR) [10]. This oxidative stress environment is further corroborated by significantly elevated markers in the peritoneal fluid, follicular fluid, and peripheral blood of patients with EMs [11–14]. An imbalance between ROS production and antioxidant detoxification (i.e., ROS scavenging by cellular and mitochondrial defense systems) results in oxidative stress, which can damage peritoneal mesothelial cells and facilitate the implantation of ectopic endometrial tissue [15]. Despite the established associations between mitochondrial dysfunction and EMs, it remains unclear whether FOXQ1, a recognized driver of cancer progression, contributes to EMs by directly modulating mitochondrial function. Consequently, our study aimed to investigate this previously unexplored connection.

Our previous study demonstrated that FOXQ1 knockdown in senescent granulosa cells significantly elevated matrix metalloproteinase (MMP) levels, enhanced ATP production, and increased mtDNA content [16], suggesting a crucial link between FOXQ1 and mitochondrial function. However, the precise relationship between FOXQ1 and mitochondrial dynamics in EMs remains to be fully elucidated and warrants further investigation to determine whether similar mechanisms operate in the pathogenesis of this disease.

Therefore, the dual objectives of this study were to: [1] mechanistically characterize FOXQ1-mediated mitochondrial dysfunction in EMs progression, and [2] identify FOXQ1 as a biomarker with diagnostic potential for endometriosis.

## Materials and methods

### Sample collection and preparation

Eutopic endometrial (*n* = 29), ovarian EMs (*n* = 27), pelvic sidewall EMs (*n* = 10) and bowel EMs (*n* = 10) tissues were collected from 76 patients at Hubei Maternal and Child Health Hospital from the biobank between November 2021 and August 2025. Participants were required to meet the following eligibility requirements: regular menstrual cycles without hormone therapy for at least one month prior to surgery and the proliferative phase of the menstrual cycle. Patients were excluded if they were diagnosed with pregnancy, cancer, active acute pelvic inflammatory disease, malignant tumors, infectious diseases, systemic diseases, or hereditary diseases. Patient age ranged from 20 to 50 years. Eutopic endometrium was collected from patients without EMs by hysteroscopic surgery, whereas ectopic endometrial tissue originates from patients with ovarian, pelvic sidewall and bowel by laparoscopic surgery. Patients in the eutopic endometrium group were diagnosed with benign gynecological diseases unrelated to endometriosis, included endometrial polyps, cervical polyps, endometrial thickening, uterine leiomyoma, ovarian serous cystadenoma, and simple ovarian cysts. EMs was diagnosed based on histological verification of both endometrial-like epithelium and stroma in lesions obtained during laparoscopic excision.

## Immunohistochemical staining

Tissue samples were fixed in 4% paraformaldehyde, embedded in paraffin and and sectioned at 4 μm thickness. After deparaffinization in xylene and rehydration through a graded ethanol series (100, 95, 85, and 75%), antigen retrieval was performed by heating in 0.01 M citrate buffer (pH 6.0) in a microwave oven for 15 min. The sections were then treated with 0.3% hydrogen peroxide for 10 min, followed by permeabilization and blocking in a buffer containing 4% donkey serum and 1% bovine serum albumin (BSA) in 1**×**PBS with Tween^®^ detergent (PBST) for 1 h at room temperature. The samples were then incubated overnight with the primary antibody (rabbit anti-human FOXQ1, IgG, 1:200; Proteintech, Wuhan, China). Subsequently, samples were incubated with universal secondary antibodies (anti-rabbit IgG, 1:10000; Proteintech, Wuhan, China) at room temperature for 30 min. The DAB (Roche, Basel, Switzerland) chromogenic agent was added dropwise, and color development was controlled under a light microscope for 10–15 min. Samples were counterstained with hematoxylin for 2 min and observed and photographed under an inverted microscope (Carl Zeiss AG, Jena, Germany).

## RT-qPCR

Total RNA was extracted using TRIzol (Vazyme, Nanjing, China) and reverse-transcribed using random primers and the PrimeScript RT reagent kit (Vazyme, Nanjing, China) according to the manufacturer’s protocol. The primers were designed and synthesized by Sangon Biotechnology Ltd. (Sangon, Shanghai, China). The primer sequences are shown in Table 1. GAPDH was used as the housekeeping gene. The reaction was performed in a total volume of 20 µL containing 1 µL of cDNA, 10 µM of forward and reverse primers, and 10 µL of 2×SYBR Green master mix. The cycling conditions used were as follows: 95 °C for 3 min and 40 cycles at 95 °C for 10 s, 60 °C for 30 s, and 72 °C for 30 s. The relative expression was calculated using the 2^−ΔΔC^t method.

**Table 1:** Primers sequence

Gene	Primers sequence
FOXQ1 forward	GGCGGAGATCAACGAGTACC
FOXQ1 reverse	GTACTCGCTGTTGGGGTTGA
IL-8 forward	ACTGAGAGTGATTGAGAGTGGAC
IL-8 reverse	AACCCTCTGCACCCAGTTTTC
IL-6 forward	ACTCACCTCTTCAGAACGAATTG
IL-6 reverse	CCATCTTTGGAAGGTTCAGGTTG
IL-1β forward	ATGATGGCTTATTACAGTGGCAA
IL-1β reverse	GTCGGAGATTCGTAGCTGGA
TNFα forward	CCTCTCTCTAATCAGCCCTCTG
TNFα reverse	GAGGACCTGGGAGTAGATGAG

## Culture of primary human endometrial epithelial cells (hEECs)

Primary human endometrial epithelial cells (hEECs) were purchased from Shanghai Hongshun Biotechnology Co., LTD (Hongshun, Shanghai, China). hEECs were cultured in DMEM/F12 medium (Hyclone, Utah, USA) containing 10% FBS v/v (Gibco, California, USA) and 1% penicptomycin (Gibco, California, USA) at 37℃ in a humidified atmosphere under 5% CO_2_.

## Cell counting Kit-8 (CCK-8) assay

The CCK-8 assay was performed according to the manufacturer’s instructions (Beyotime, Shanghai, China). In brief, hEECs were inoculated into each well of a 96-well plate at a density of 3 × 10³ cells in 100 µL of complete growth medium (DMEM/F12 medium supplemented with 10% FBS and 1% penicptomycin), and each group had was tested in triplicate. Following reverse transfection, cells in the experimental group were subjected to either FOXQ1 overexpression (FOXQ1 OE) or FOXQ1 knockdown (siFOXQ1). The corresponding control groups were treated with an empty vector or a scrambled control siRNA, respectively. After incubation for 48 h, 10 µL of the CCK8 reagent (Yeasen, Shanghai, China) was added to each well, followed by further incubation in an incubator for 4 h. Subsequently, the absorbance at 450 nm was immediately measured using Synergy HTX Multi-Mode Reader (BioTek, Vermont, USA) to calculate the proliferative potential and normalize with respect to the control, according to the formula (%)=(average value of experimental group-blank control group)/(average value of control group-blank control group)×100%.

### Fluorescence-activated cell sorting (FACS) analysis

The cell cycle was detected using FACS (BD Biosciences, New Jersey, USA). Cells were stained with a solution containing 10 µL of propidium iodide (PI, Bestbio, Shanghai, China) and 5 µL of Annexin V-FITC (Bestbio, Shanghai, China), followed by incubation in the dark at room temperature for 5–15 min. Green fluorescence from Annexin V-FITC was measured using the FITC channel (FL1), and red fluorescence from PI was measured using the PI channel (FL2). The flow cytometry parameters were as follows: excitation wavelength, Ex = 488 nm; emission wavelength, Em = 530 nm. The fluorescently labeled cells were treated with a staining working solution containing 500 µL PI/RNase A at room temperature while avoiding light exposure for 30–60 min. Red fluorescence was recorded using a computer at an excitation wavelength of 488 nm.

## Western blotting

Total protein was extracted using RIPA buffer, and the protein concentration was determined using a BCA Protein Assay Kit (Beyotime, Shanghai, China). The samples were denatured by boiling for 5 min and separated on a 12% sodium dodecyl sulfate-polyacrylamide gel electrophoresis gel. The proteins were then transferred to a PVDF membrane, blocked with 5% (w/v) skim milk for 1 h, and incubated with primary antibodies, including rabbit anti-FOXQ1 (1:2000; Abcam, UK) and mouse anti-GAPDH (1:6000; Abcam, UK). The membrane was then incubated with a secondary antibody (anti-rabbit IgG, 1:10000 and anti-mouse IgG, 1:10000, respectively; Proteintech, Shanghai, China) for 1 h at room temperature. The immunoreactive bands were detected by chemiluminescence (Beyotime, Shanghai, China) according to the manufacturer’s instructions. The chemiluminescent signals were then visualized and digitally captured using a ChemiDoc XRS+ Chemiluminescence Gel Imaging System (Bio-Rad, California, USA).

## EdU detection

Cells transfected on climbing slices were fixed with 4% paraformaldehyde in PBS. After fixing, 250 µL of 2 mg/mL glycine was added to each well, and the cells were incubated for 5 min in a shaking incubator to decolorize. The glycine solution was then removed. Cells were washed with 500 µL PBS for 5 min, and the PBS was aspirated off. Subsequently, 500 µL of 0.5% Triton X-100 in PBS was added for permeabilization for 10 min. Following a single PBS wash for 5 min, cells were stained with 500 µL of 1× Apollo (RiboPharm‌, Guangzhou, China) and 500 µL of 1× Hoechst 33,342 solutions (Sigma-Aldrich, Missouri, USA) in a shaking incubator for 30 min. The stained cells were covered with an anti-fade reagent (RiboPharm‌, Guangzhou, China) and imaged under a LSM 900 confocal microscope (Carl Zeiss AG, Jena, Germany).

### TUNEL experiment

The TUNEL assay was performed using the In Situ Cell Death Detection Kit, Fluorescein (Roche, Basel, Switzerland) according to the manufacturer’s instructions. Transfected cells were individually placed in environmentally transparent agents (three cylinders for 10 min each) and gradient alcohol (anhydrous, 95%, 75%, each cylinder for 5 min), rinsed with tap water, and soaked in distilled water. Subsequently, 0.1% TritonX 100 and 0.1% sodium citrate were added dropwise at room temperature for 8 min. The slices were then washed with 1× TBS for 5 min. To block nonspecific reactions, each slice was then incubated for 30 min at room temperature with a combination of 3% BSA and 20% normal calf serum. After discarding the serum, each slice was treated with a TUNEL reaction mixture. The slices were incubated in a humidity box at 37℃ while the working solution was replaced by 1× TBS as negative control for 1 h. Following three rounds of washing with 1× TBS for 5 min each, DAPI staining was performed at room temperature under light-shielding conditions for 10 min. Finally, specimens were sealed with anti-fluorescence attenuation tablets and photographed using a LSM 900 confocal microscope (Carl Zeiss AG, Jena, Germany).

### Wound healing assay

hEECs were transfected with siFOXQ1 or FOXQ1 OE in 6-well plates at a density of 3 × 10^5^ cells per well and cultured for 48 h prior to further analysis. Two scratches were made on the cell layer perpendicular to the cell plane using a pipette tip. Once the scratches were complete, the cells were washed three times with 1× PBS, and the medium was replaced with serum-free medium. The plates were then incubated at 37℃ in a 5% CO_2_ incubator. At 0 and 24 h, the width of the wound was observed and photographed under a microscope in the corresponding visual field (Carl Zeiss AG, Jena, Germany).

### Transwell invasion/migration experiment

The basement membrane was coated on ice, the Matrigel matrix (ABW, Shanghai, China) was homogenized using a precooled liquid transfer gun, and the Matrigel glue was diluted in a serum-free medium (8:1). Diluted matrix glue was added vertically to the upper chamber of a 24-well Transwell plate at a volume of 60 µL per well, ensuring complete coverage. The plate was then incubated overnight at 37℃ to allow binding of the matrix glue. Unbound matrix glue was removed by washing the cells with serum-free medium. Excess liquid from the culture plate was aspirated, and 50 µL of serum-free culture medium containing 10 g/L BSA was added to each well. After an additional incubation period of 30 min in a 37℃ incubator, the inoculated cells were cultured in a separate 24-well plate for 48 h. Subsequently, the Transwell chamber was carefully removed, and any remaining culture medium was absorbed. For fixation, cells were treated with 4% paraformaldehyde for 20 min and washed thrice with PBS. The Transwell board was then removed and air-dried. Staining with a solution of crystal violet (0.1%) lasted for approximately 20 min before washing three times with PBS. Non-specific staining on the upper side of the chamber walls was gently wiped away using cotton swabs prior to microscopic examination (Carl Zeiss AG, Jena, Germany).

### Mitochondrial function detection

Methods for Mitochondrial function detection (such as mitochondrial DNA (mtDNA), ATP and ROS levels) were referenced in the article [17].

### QPCR detection of mtDNA levels

DNA was extracted using an Allprep Cell RNA/DNA Mini Kit (QIAGEN, Düsseldorf, Germany) according to the manufacturer’s protocol. Briefly, cells were first trypsinized with 500 µL of Trypsin EDTA, incubated for 3–5 min, resuspended in 600 µL of lysis buffer, mixed thoroughly by pipetting, and homogenized using a 1 mL syringe using a blunt needle. The lysate was applied to a DNA-binding column and centrifuged at 14,000 rpm for 60 s to capture the DNA. The flow-through was then discarded. The column was then washed with 500 µL of inhibitor removal solution, centrifuged at 12,000 rpm for 30 s, and the flow-through was discarded. This wash step was repeated with 700 µL of wash buffer, followed by a second wash with an additional 500 µL of wash buffer. DNA was eluted by centrifuging the column in a fresh tube at 13,000 rpm for 2 min after the addition of 100 µL of elution buffer to the column membrane. The column was incubated with elution buffer for 3–5 min, then centrifuged at 12,000 rpm for 1 min to collect purified DNA.

### ATP levels

Relative ATP content was measured using an ATP Assay Kit (Beyotime, Shanghai, China) according to the manufacturer’s protocol. Briefly, 200 µL of lysate was added to each well of a 6-well plate or tissues. Following centrifugation at 12,000*×*g for 5 min at 4℃ after pyrolysis, the resulting supernatant was collected for subsequent determination. The reagent was dissolved on ice, and the ATP standard solution was diluted to an appropriate concentration gradient for ATP detection. The relative light unit (RLU) value was measured using a Synergy HTX Multi-Mode Reader (BioTek, Vermont, USA).

### ROS assay

ROS levels were measured using a ROS assay kit with a DCFH-DA probe (Beyotime, Shanghai, China) according to the manufacturer’s protocol. Briefly, hEECs were rinsed and incubated with the fluorescent probe at 37℃ for 25 min in the dark. Cells were then examined using LSM 900 confocal microscope (Carl Zeiss AG, Jena, Germany).

### Seahorse assay

Mitochondrial function was measured using the Seahorse XF Cell Mitochondrial Pressure Test Kit (Agilent, California, USA) according to the manufacturer’s protocol.

### Mitochondrial membrane potential

The mitochondrial markers of living cells were stained by Mito-Tracker Red CMXRos (Beyotime, Shanghai, China), according to the manufacturer’s protocol. Briefly, hEECs were incubated with Mito-Tracker Red CMXRos staining working fluid at 37℃ for 20 min.

### Statistical analysis

Data are displayed as the Mean ± SD. Statistical analyses and chart generation were performed using the Statistical Package for Social Sciences (Version 12.0; SPSS Inc., Chicago, IL, USA). The normality of continuous variables between the two groups was assessed, and the homogeneity of variance was tested using t-test. In cases where the variance was unequal, a corrected t-test was applied. The expression rate between the two groups was compared using the χ² test, while nonparametric statistics such as Spearman correlation were employed to analyze correlations. Comparisons between multiple groups were performed using a one-way ANOVA. A receiver operating characteristic (ROC) curve analysis was performed to assess the diagnostic value for EMs. P value of < 0.05 was considered statistically significant.

## Results

### Description of the study population

In this study, general clinical characteristics of the control and EMs groups were analyzed. No significant differences were observed between the two groups in terms of age, body mass index (BMI), gravidity, or parity (*P* > 0.05; Table 2).

**Table 2:** Comparison of general data between two groups of patients

Characteristic	Control group(*n* = 29)	EMs group(*n* = 27)	t	*P*
Age (years)	29.57 ± 4.69	30.67 ± 5.59	-0.73	0.47
BMI (kg/m^2^)	20.32 ± 4.19	20.49 ± 1.99	0.18	0.86
Gravidity (number)	2.52 ± 0.79	2.21 ± 1.17	-2.34	0.06
Parity (number)	2.13 ± 1.29	1.46 ± 1.47	1.66	0.10

Data are displayed as the Mean ± SD

### Correlation between FOXQ1 mRNA expression level and clinical parameters in patients with EMs

The age, clinical stage, and cyst diameter of patients in the EMs group were further evaluated, and the correlations between these clinical parameters and FOXQ1 mRNA expression levels were analyzed. As shown in Table 3, FOXQ1 mRNA expression was not significantly correlated with age, clinical stage, or cyst diameter in patients with EMs.

**Table 3:** Analysis of correlation between the FOXQ1 mRNA level and clinical parameters in EMs

		Number of cases(*n* = 27)	Correlation coefficient	P
Age (years)	20–29	11	−0.028	0.891
	30–39	12		
	40–49	4		
Staging	Ⅱ	1	−0.112	0.578
	Ⅲ	11		
	Ⅳ	15		
Lesions Diameter(cm)	≥ 5	14	0.082	0.689
	<5	13		

### ROC curve

To assess the diagnostic value of FOXQ1 in EMs, FOXQ1 mRNA expression levels were used as the test variable to construct a receiver operating characteristic (ROC) curve. As shown in Figure S1, the area under the curve (AUC) for FOXQ1 in diagnosing EMs was 0.8784 (*P* < 0.0001), suggesting that FOXQ1 may serve as a potential diagnostic biomarker for EMs.

#### FOXQ1 level in ectopic endometrium of EMs

**Fig. 1:**
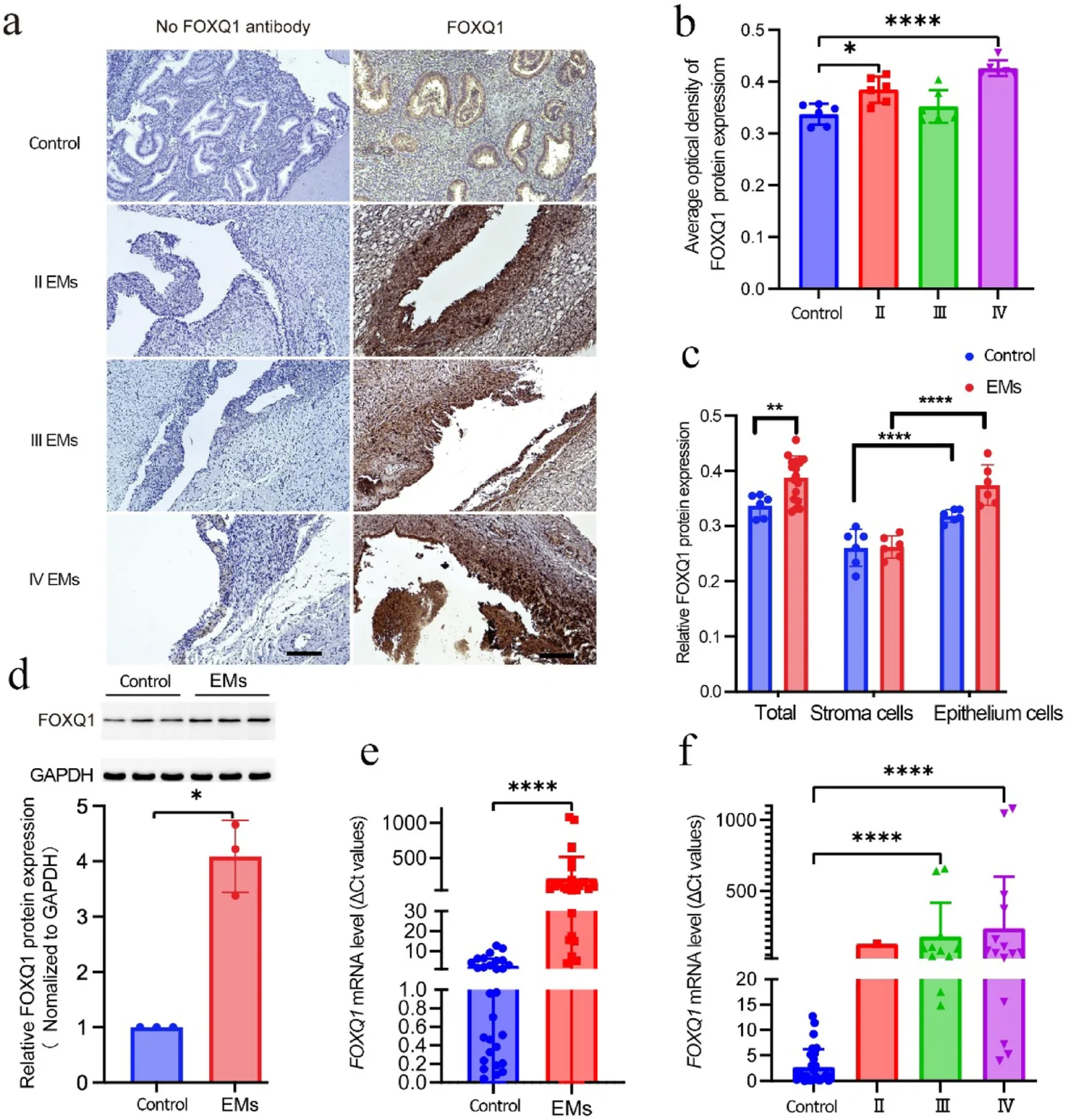
FOXQ1 is upregulated in EMs. **a** Representative immunohistochemical (IHC) staining images of FOXQ1 in EMs and control tissues (*n* = 24). Scale bar, 200 μm. **b** Comparison of FOXQ1 protein levels in different stages of EMs group, *n* = 24. **c** Relative FOXQ1 protein levels in stromal and epithelial cells after normalization to the control group. **d** Comparison of FOXQ1 protein levels between EMs group and control group, *n* = 3. **e** Detection of FOXQ1 mRNA level (ΔCt values) by RT-qPCR, Control group, *n* = 29. EMs group, *n* = 27. **f** Detection of FOXQ1 mRNA level (ΔCt values) between control group and EMs groups with different stages by RT-qPCR, Control group, *n* = 29. EMs group, *n* = 27. Lower ΔCt values indicate higher relative expression. *P < 0.05, **P < 0.01, *** *P* < 0.001, **** *P* < 0.0001. Normality of continuous variables between the two groups was assessed, and homogeneity of variance was tested using a two independent samples t-test; in cases where variance was unequal, a corrected t-test was utilized instead

Immunohistochemistry was performed to detect FOXQ1 protein expression in both the EMs and control groups. As shown in Fig. 1, FOXQ1 protein expression was detected in both epithelial and stromal cells within ectopic endometriotic lesions, with stronger staining intensity observed in epithelial cells (Fig. 1a and c). Therefore, endometrial epithelial cells were selected for subsequent experiments.

The results demonstrated that FOXQ1 protein expression was significantly higher in the EMs group than in the control group, with variations observed across different disease stages (Fig. 1b). Western blot analysis was conducted to further quantify FOXQ1 protein expression, and differences between groups were analyzed using ImageJ software (Fig. 1d). The EMs group exhibited a significantly higher level of FOXQ1 protein expression compared with the control group (*P* < 0.05).

Consistently, FOXQ1 mRNA expression was significantly elevated in the EMs group compared with the control group (*P* < 0.0001; Fig. 1e). Furthermore, within the EMs group, FOXQ1 mRNA levels were higher across different clinical stages than those in the control group, indicating a positive association with disease progression (Fig. 1f).

We further evaluated FOXQ1 mRNA expression in pelvic sidewall and bowel EMs. The results demonstrated that, consistent with ovarian EMs, FOXQ1 expression was significantly upregulated in both pelvic sidewall and bowel EMs compared with the control group (Figure S2).

#### FOXQ1 promotes proliferation and inhibitor apoptosis in the hEECs

**Fig. 2:**
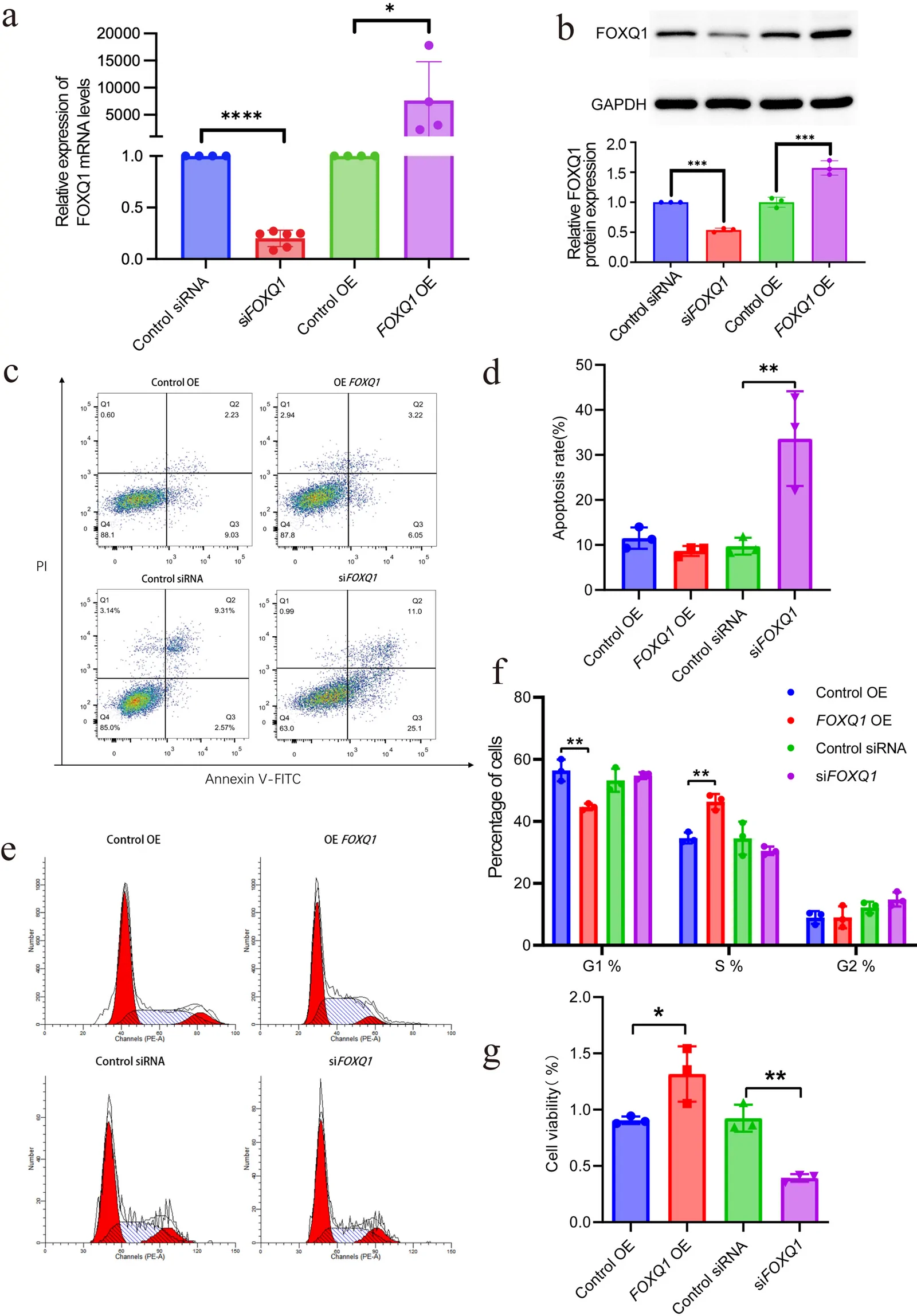
FOXQ1 promotes proliferation and inhibitor apoptosis in the hEECs. **a** and **b**. The relative expression level of FOXQ1 in FOXQ1 OE and siFOXQ1 groups were detected using RT-qPCR and western blot. **c** and **d**. Flow cytometry detect cells apoptosis. **e** and **f**. Flow cycle detection. **g**. CCK8 cell proliferation assay. *P <0.05, *** P <0.001, **** P <0.0001. Each experiment was performed at least three times. The normality of continuous variables between the two groups was assessed, and homogeneity of variance was tested using a two-independent samples t-test. In cases where variance was unequal, a corrected t-test was applied.

To verify transfection efficiency, RT-qPCR and western blot analyses were performed in the FOXQ1 overexpression (FOXQ1 OE) and knockdown (siFOXQ1) groups. As shown in Fig. 2a and b, both mRNA and protein levels of FOXQ1 were significantly increased in the FOXQ1 OE group compared with the control group (*P* < 0.0001). In contrast, FOXQ1 mRNA and protein levels were significantly reduced in the siFOXQ1 group compared with the control group (*P* < 0.0001).

In the present study, five independent assays were employed to evaluate the effects of FOXQ1 on hEECs proliferation and apoptosis. Flow cytometric analysis (Fig. 2c and d) showed that, compared with the control group, the FOXQ1 OE group exhibited a decrease in apoptosis rate that did not reach statistical significance (*P* > 0.05), whereas the siFOXQ1 group demonstrated a significant increase in apoptosis rate (*P* < 0.01).

Cell cycle analysis by flow cytometry (Fig. 2e and f) revealed that FOXQ1 OE significantly increased the proportion of cells in the S phase and decreased the proportion in the G1 phase (*P* < 0.01), indicating enhanced DNA synthesis through promotion of S-phase entry.

**Fig. 3:**
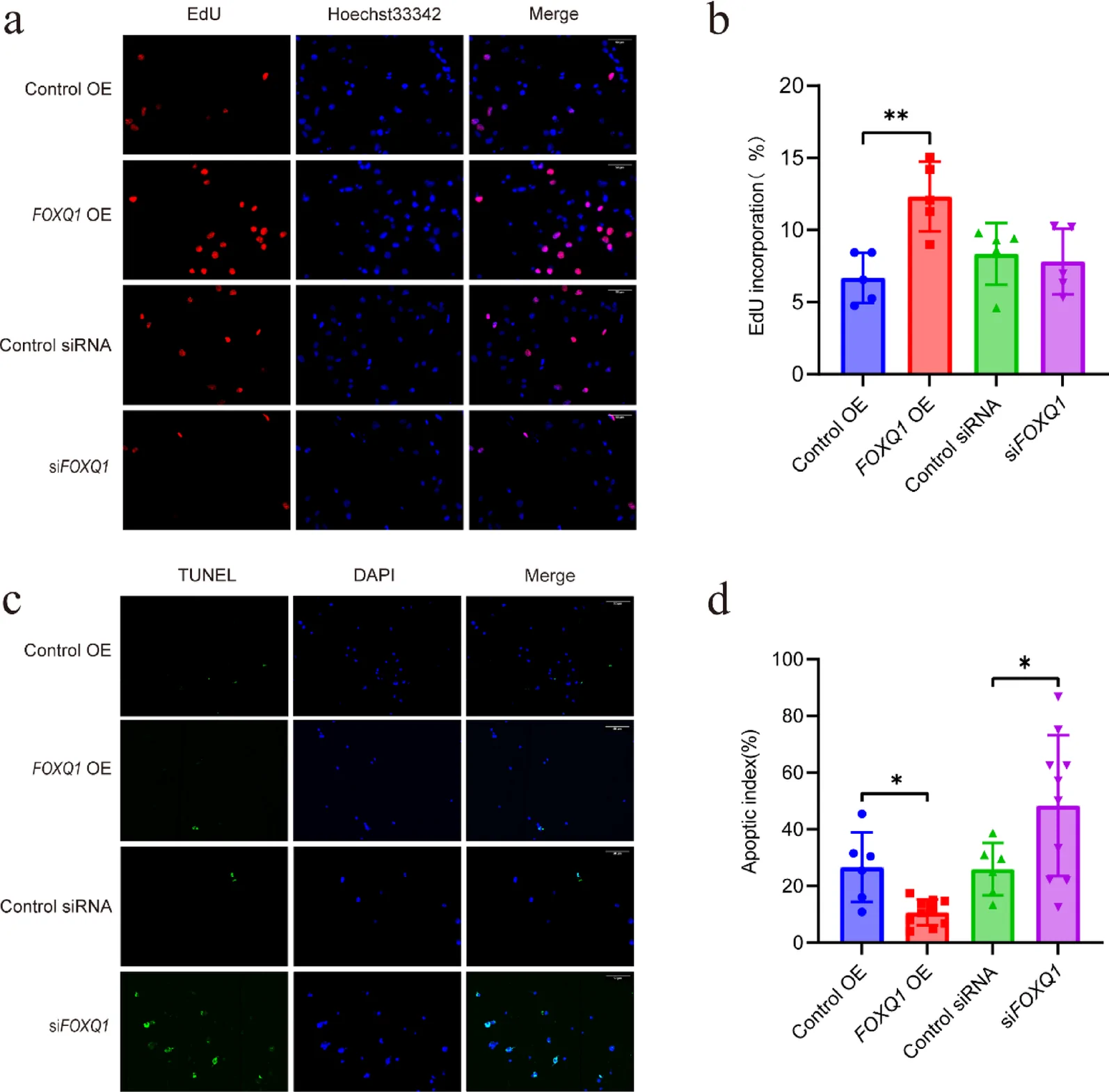
FOXQ1 promotes proliferation in the hEECs. **a** Representative images of the EdU cell proliferation assay. Scale bar, 50 μm. **b** Quantification of the EdU assay. Data are presented as the fold change relative to the control group after normalization. **c** Representative images of the TUNEL apoptosis assay. Scale bar, 50 μm. **d** Quantification of the TUNEL assay. Data are presented as absolute values (percentage of positive cells) for direct comparative analysis between groups. Each experiment was performed at least three times. Normality of continuous variables between the two groups was assessed, and homogeneity of variance was tested using t-test; in cases where variance was unequal, a corrected t-test was applied

The CCK-8 assay results (Fig. 2g) demonstrated that FOXQ1 OE significantly enhanced proliferative capacity compared with the control group (*P* < 0.05), whereas proliferation was significantly reduced in the siFOXQ1 group (*P* < 0.01). Consistently, the EdU assay (Fig. 3a and b) showed that proliferation was significantly increased in the FOXQ1 OE group (*P* < 0.01), while the siFOXQ1 group showed a decrease in proliferation that did not reach statistical significance (*P* > 0.05).

TUNEL staining (Fig. 3c and d) further demonstrated that the apoptosis index was significantly decreased in the FOXQ1 OE group (*P* < 0.05) and significantly increased in the siFOXQ1 group (*P* < 0.05).

#### FOXQ1 promotes hEECs invasion and migration

**Fig. 4:**
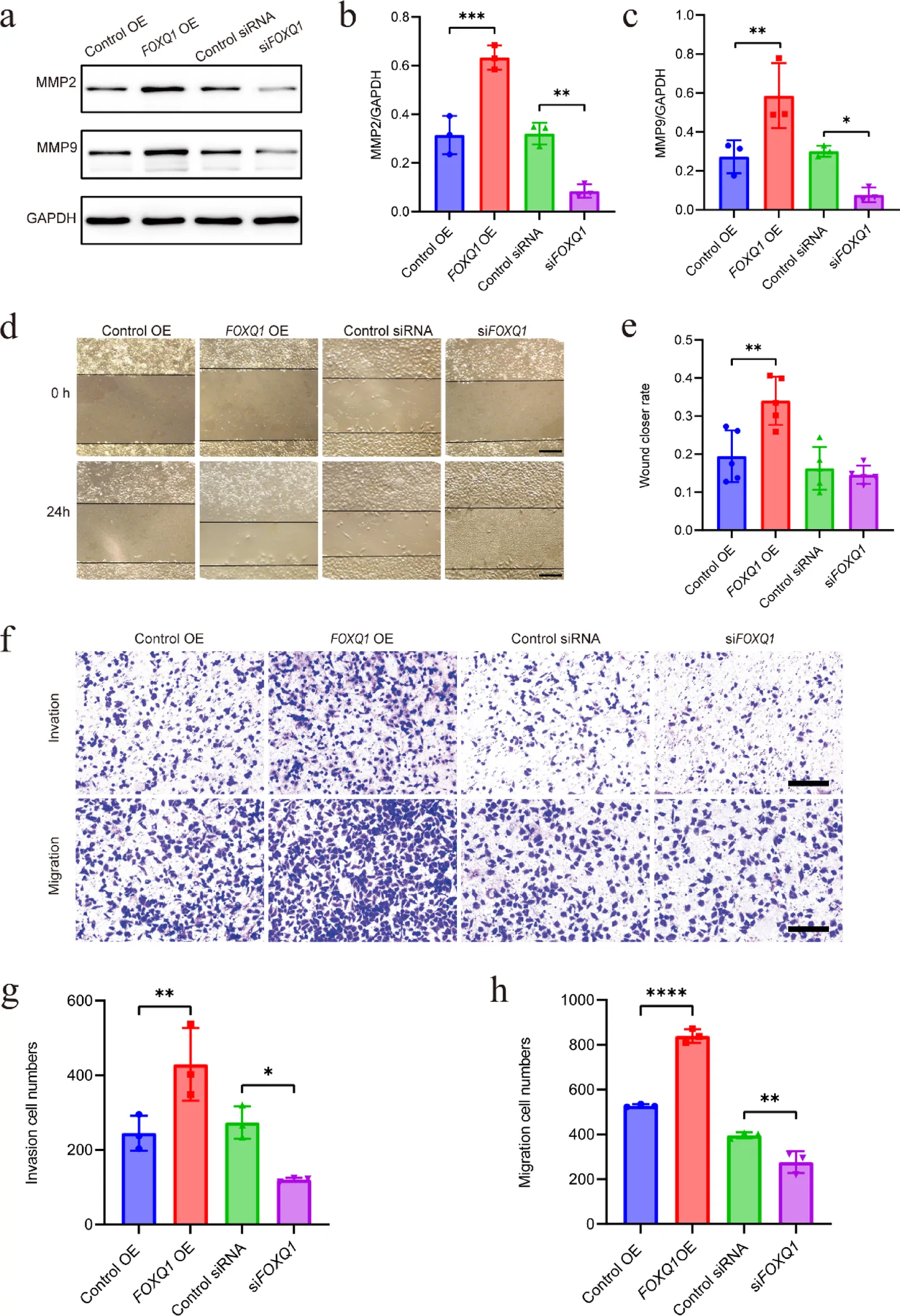
FOXQ1 promotes hEECs invasion and migration. **a** The expressions of MMP2 and MMP9 detected using western blot analysis. **b** Graphical plot of the MMP2 protein expression. **c** Graphical plot of the MMP9 protein expression. **d** Scratch test. **e **Wound closer rate. **f ** Transwell experiment. **g **Invasion cell numbers. **h** Migration cell numbers. *P < 0.05, **P < 0.01, ***P < 0.001, ****P < 0.0001. Scale bar, 100 μm. Each experiment was performed at least three times. The normality of continuous variables between the two groups was assessed, and homogeneity of variance was tested using a two-independent samples t-test; in cases where variance was unequal, a corrected t-test was applied

Next, we investigated the effects of FOXQ1 on hEEC invasion and migration. Western blot analysis showed that the protein expression of MMP2, which is associated with invasion and migration, was significantly higher in the FOXQ1 OE group than in the control group (*P* < 0.001; Fig. 4a and b). Conversely, MMP2 expression was significantly reduced in the siFOXQ1 group compared with the control group (*P* < 0.01).

Similarly, MMP9 protein expression followed the same trend as MMP2, with significantly higher levels in the FOXQ1 OE group (*P* < 0.01; Fig. 4a and c) and significantly lower levels in the siFOXQ1 group compared with the control group (*P* < 0.05).

The wound healing assay (Fig. 4d and e) demonstrated that, at 24 h after scratching, the migration capacity of cells in the FOXQ1 OE group was significantly greater than that in the control group (*P* < 0.01). Although migration appeared reduced in the siFOXQ1 group compared with the control group, the difference was not statistically significant (*P* > 0.05). These results suggest that FOXQ1 overexpression promotes hEECs migration.

Furthermore, the Transwell invasion assay revealed that the number of cells penetrating the membrane was significantly higher in the FOXQ1 OE group than in the control group (*P* < 0.01; Fig. 4f and g), whereas significantly fewer penetrating cells were observed in the siFOXQ1 group compared with the control group (*P* < 0.05).

Similarly, in the migration assay, the FOXQ1 OE group exhibited a significantly greater number of membrane-penetrating cells compared with the control group (*P* < 0.0001; Fig. 4f and h), while the siFOXQ1 group showed significantly fewer migrating cells than the control group (*P* < 0.01). Collectively, these findings indicate that FOXQ1 enhances hEECs invasion and migration.

### FOXQ1 promotes mitochondrial function in the hEECs

**Fig. 5:**
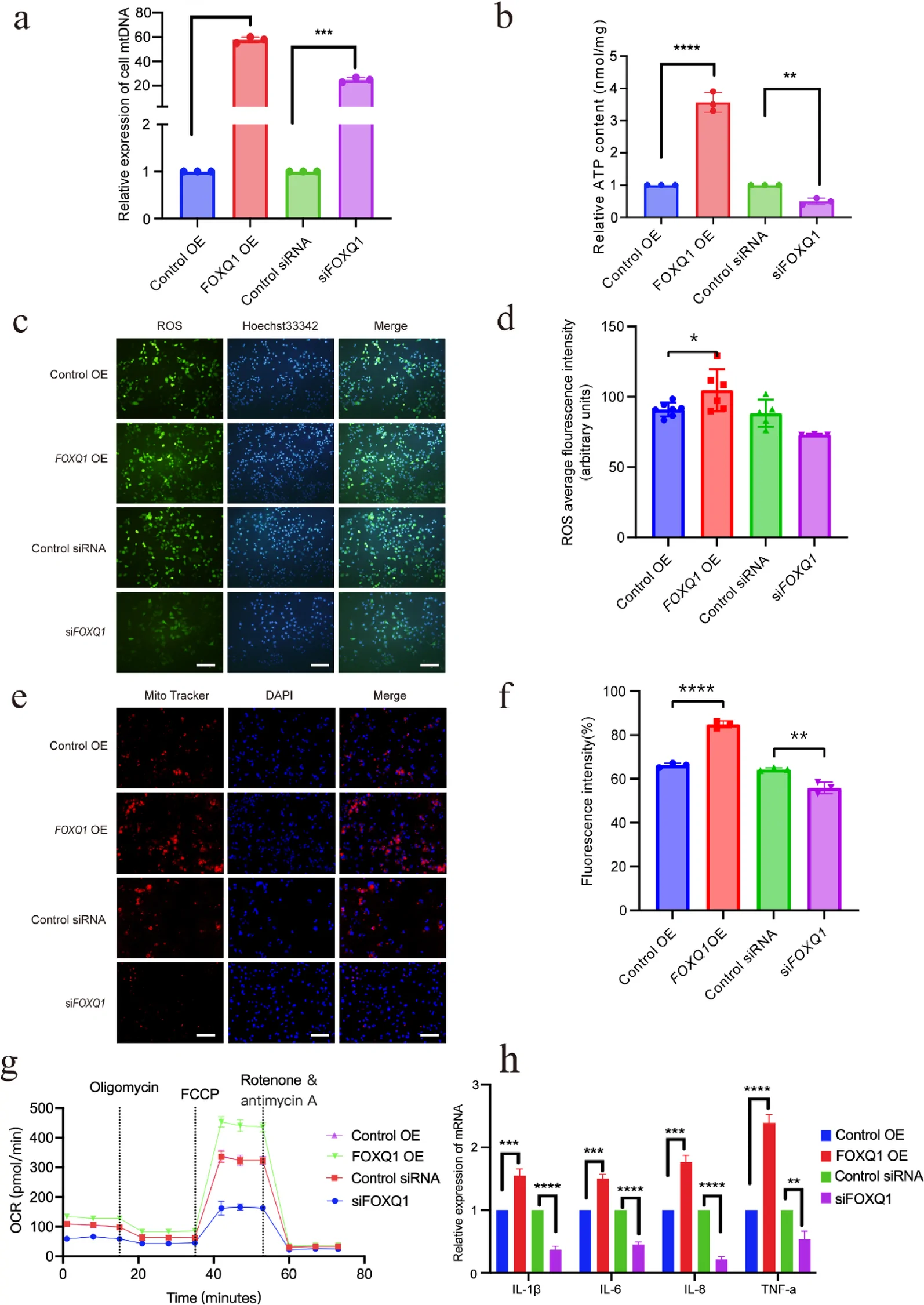
FOXQ1 promotes mitochondrial function in hEECs. **a** the mtDNA expression; **b** the mitochondrial ATP level; **c** ROS detection; **d** the average fluorescence intensity of ROS; **e** MitoTracker detection; **f** the average fluorescence intensity of MitoTracker; **g** OCR; **h** the expression of IL-1β, IL-6, IL-8 and TNF-α.*P < 0.05, **P < 0.01, ***P < 0.001, ****P < 0.0001. Scale bar, 100 μm. Each experiment was performed at least three times. The normality of continuous variables between the two groups was assessed, and homogeneity of variance was tested using a two-independent samples t-test; in cases where variance was unequal, a corrected t-test was applied

QPCR analysis was performed to quantify relative mtDNA expression levels (Fig. 5a). The FOXQ1 OE group exhibited significantly higher mtDNA levels compared with the control group (*P* < 0.05). Although mtDNA levels were reduced in the siFOXQ1 group compared with the control group, the difference did not reach statistical significance (*P* > 0.05), and the potential biological implications warrant further investigation.

ATP measurement (Fig. 5b) showed that ATP levels were significantly increased in the FOXQ1 OE group compared with the control group (*P* < 0.05), whereas ATP levels were significantly decreased in the siFOXQ1 group (*P* < 0.05).

Fluorescence intensity analysis of intracellular ROS (Fig. 5c and d) demonstrated that ROS levels were significantly elevated in the FOXQ1 OE group compared with the control group (*P* < 0.05) and significantly reduced in the siFOXQ1 group (*P* < 0.05).

In addition, MitoTracker staining intensity and oxygen consumption rate (OCR) were significantly increased in the FOXQ1 OE group compared with the control group (Fig. 5e–g; *P* < 0.05), whereas these effects were reversed in the siFOXQ1 group.

Collectively, these findings suggest that FOXQ1 modulates mitochondrial function by enhancing cellular energy production and oxygen consumption.

### FOXQ1 promotes the mRNA expression of inflammatory cytokines in the hEECs

RT-qPCR analysis revealed that the mRNA expression levels of interleukin (IL)-6, IL-1β, tumor necrosis factor (TNF)-α, and IL-8 (Fig. 5h) were significantly increased in the FOXQ1 OE group compared with the control group (*P* < 0.05). Conversely, the siFOXQ1 group exhibited significantly reduced mRNA expression levels of IL-6, IL-1β, TNF-α, and IL-8 compared with the control group (*P* < 0.05).

## Discussion

The etiology of EMs is complex and shares numerous characteristics with malignant tumors, as both conditions exhibit cellular proliferation, invasion, migration, and distant metastasis. They are also characterized by extensive metabolic reprogramming, including excessive glucose uptake, aerobic glycolysis, high lactate production, and alterations in energy metabolism [18]. However, EMs remains a benign gynecological disorder. Therefore, we selected FOXQ1, a well-established oncogenesis-promoting factor, to explore its potential role in the pathogenesis of EMs. In the present study, we validated the differential expression of FOXQ1 in EMs tissues across various disease stages and analyzed its correlations with patient age, endometriotic cyst stage, and cyst diameter. Additionally, we assessed the diagnostic value of FOXQ1 for predicting EMs.

First, we examined the association between FOXQ1 expression level and clinical parameters in patients with EMs. Spearman’s correlation analysis revealed no significant correlations between FOXQ1 expression and age, endometriotic cyst diameter, or EMs stage, suggesting that neither patient age nor cyst diameter significantly influenced FOXQ1 expression in tissue samples. Notably, our previous RT-qPCR analysis comparing FOXQ1 expression in ectopic endometrial tissues from patients at different stages demonstrated that relative expression increased with disease progression. The discrepancy between these findings may be attributable to the limited sample size in the clinical correlation analysis, highlighting the need to expand the sample size in future studies to improve statistical power. Furthermore, ROC curve analysis demonstrated that FOXQ1 exhibited high predictive efficiency for diagnosing EMs (AUC = 0.8784; *P* < 0.0001), indicating that FOXQ1 may serve as a potential invasive biomarker for EMs. This finding is consistent with the study by Yang et al., which reported significant prognostic value for this indicator [19]. The use of patient-derived tissue samples is rational for identifying EMs-related biomarkers. Current approaches for EMs diagnosis include non-invasive methods, such as the detection of glycoproteins, cytokines, immune cell populations, miRNAs, transcriptomic, proteomic, and metabolomic signatures in bodily fluids, as well as invasive approaches, including endometrial biopsy and assessment of epigenetic markers [20]. However, the lack of standardized protocols for sample collection, storage, and transportation has hindered biomarker discovery in EMs. Future studies should implement standardized procedures for biological sample handling to facilitate the identification of reliable diagnostic biomarkers.

We further observed that FOXQ1 expression was significantly upregulated in ectopic endometrial tissues from patients with EMs compared with eutopic endometrial tissues from non-EMs patients. More importantly, FOXQ1 OE significantly reduced apoptosis in the hEECs while enhancing their proliferation, invasion, and migration. These findings are consistent with previous observations in tumor biology. As an oncogenesis-promoting factor, FOXQ1 is frequently upregulated in various cancers and contributes to enhanced proliferation, invasion, distant metastasis, and chemoresistance. Chen et al. demonstrated that FOXQ1, as a downstream effector of the JAK2/STAT3 signaling pathway, plays a critical role in regulating epithelial–mesenchymal transition (EMT), invasion, and migration [21]. Moreover, FOXQ1 downregulation has been shown to arrest the cell cycle at the G1/S transition, thereby inhibiting cell proliferation and reducing motility and invasiveness [22]. These findings suggest that FOXQ1 may contribute to the pathogenesis of EMs.

In the present study, we found that FOXQ1 OE significantly increased the expression of MMP2 and MMP9. These matrix metalloproteinases are key extracellular enzymes that degrade extracellular matrix components and are critically involved in promoting invasion and migration of EMs cells [23]. The pro-invasive and pro-migratory effects of FOXQ1 in endometriotic cells may therefore be mediated through upregulation of MMP2 and MMP9. Recent studies have further highlighted the role of MMPs in endometriosis pathogenesis, with patients exhibiting elevated expression levels of MMP2 and MMP9 [24]. Increased metalloproteinase activity in the endometrium of women with endometriosis may promote degradation of the peritoneal extracellular matrix, thereby facilitating implantation and establishment of ectopic lesions within the peritoneal cavity [25]. Nevertheless, the precise molecular mechanisms underlying FOXQ1-mediated regulation of MMP expression warrant further investigation.

Mitochondria play a central role in cellular bioenergetics and metabolism, serving as primary sites for ATP production and ROS generation [26]. The mtDNA exists in multiple copies per cell and is highly susceptible to oxidative damage, which may impair replication and transcription, thereby affecting mitochondrial function and ROS production [27]. Emerging evidence suggests that mitochondrial dysfunction is closely associated with altered biological characteristics of endometriotic lesions. Ectopic endometrial mitochondria are exposed to chronic oxidative stress and may upregulate biosynthetic pathways to enhance energy production as an adaptive response to hypoxia [28]. Additionally, dysregulation of pro- and anti-apoptotic factors within the mitochondria-mediated apoptotic pathway may promote survival and implantation of ectopic endometrial cells [29]. However, current understanding of mitochondrial alterations in ectopic hEECs and the potential regulatory role of FOXQ1 in mitochondrial function during EMs progression remains limited. In this study, FOXQ1 OE increased mitochondrial membrane potential, mitochondrial respiratory activity, and mtDNA expression, leading to enhanced ATP and ROS production. These findings are consistent with previous reports demonstrating abnormal mitochondrial function and elevated ATP and ROS levels in endometriotic tissues [10]. Similar to tumor cells, endometrial cells exhibit progressive ROS accumulation over time [10, 30].Ovarian endometrioma cysts contain elevated levels of heme, iron, and hemoglobin [31]. Increased heme concentrations may enhance mitochondrial oxidative phosphorylation and ATP production in endometrial cells [32]. Therefore, the FOXQ1 OE-induced increases in ATP and ROS levels in the hEECs may be associated with enhanced oxidative metabolism and heme-related mitochondrial activation. Moreover, ectopic endometrial stromal cells (ESCs) reportedly enhance their metabolic capacity through mitochondrial morphological alterations, cristae remodeling, increased mitochondrial abundance, and higher matrix density. In addition, mitochondria in ectopic cells shift from primarily relying on oxidative phosphorylation toward greater dependence on glycolysis to meet biosynthetic demands [10, 33]. This metabolic reprogramming can further elevate ROS production [34], imposing stress on the respiratory chain. Furthermore, lactate accumulation resulting from enhanced glycolysis may create a microenvironment conducive to migration, invasion, and angiogenesis of ectopic endometrial cells [35]. Therefore, we speculate that FOXQ1 may exert bidirectional regulatory effects on mitochondrial function in ectopic endometrial cells, and the underlying molecular mechanisms require further clarification.

Our results also demonstrated that FOXQ1 OE upregulated the expression of pro-inflammatory cytokines in hEECs. These findings provide in vitro evidence supporting a potential role for FOXQ1 in establishing an inflammatory microenvironment in vivo, consistent with the inflammatory hypothesis of EMs pathogenesis. Inflammation is a central component of EMs pathophysiology and contributes to both local and systemic manifestations of the disease. EMs is associated with elevated levels of TNF-α, IL-1β, IL-6, angiogenic factors such as vascular endothelial growth factor (VEGF), growth factors, and adhesion molecules [36]. Importantly, women with EMs exhibit increased circulating levels of pro-inflammatory cytokines [37], which are significantly reduced following laparoscopic excision of lesions [38]. However, it remains unclear whether inflammation acts as a primary driver of lesion formation or represents a secondary consequence of disease progression, necessitating further in-depth investigation.

In summary, FOXQ1 overexpression may promote EMs progression by enhancing mitochondrial function in the hEECs, thereby facilitating proliferation, invasion, and migration.

## Conclusions

FOXQ1 is highly expressed in EMs tissues, and its expression increases with advancing disease stage. Moreover, FOXQ1 overexpression enhances mitochondrial function and subsequently promotes the proliferation, invasion, and migration of hEECs, thereby contributing to EMs development.

## Supplementary Information

Below is the link to the electronic supplementary material.

Supplementary Material 1

Supplementary Material 2

## Data Availability

Not applicable.

## Abbreviations

EMs: Endometriosis
RT-qPCR: Reverse transcription-quantitative PCR
mtDNA: Mitochondrial DNA
ROS: Reactive oxygen species
OCR: Oxygen consumption rate
hEECs: Human endometrial epithelial cells
MMP: Matrix metalloproteinase
FACS: Fluorescence-activated cell sorting
ROC: Receiver operating characteristic
ESCs: Ectopic endometrial stromal cells
